# Avian Influenza A Virus (H5N1) Outbreaks, Kuwait, 2007

**DOI:** 10.3201/eid1406.080056

**Published:** 2008-06

**Authors:** Ahmad Al-Azemi, Justin Bahl, Sameer Al-Zenki, Yousif Al-Shayji, Sami Al-Amad, Honglin Chen, Yi Guan, J.S. Malik Peiris, Gavin J.D. Smith

**Affiliations:** * Kuwait Institute for Scientific Research, Kuwait City, Kuwait; † University of Hong Kong, Pokfulam, Hong Kong Special Administrative Region (SAR), People’s Republic of China; ‡ The HKU-Pasteur Research Centre, Pokfulam, Hong Kong SAR

**Keywords:** highly pathogenic avian influenza, genotype Z, Middle East, virus evolution, molecular epidemiology, dispatch

## Abstract

Phylogenetic analysis of influenza A viruses (H5N1) isolated from Kuwait in 2007 show that (H5N1) sublineage clade 2.2 viruses continue to spread across Europe, Africa, and the Middle East. Virus isolates were most closely related to isolates from central Asia and were likely vectored by migratory birds.

Highly pathogenic avian influenza (HPAI) virus (H5N1) has been endemic in poultry in Asia since 2003 (*1*,*2*). From 2002 through 2005, influenza virus (H5N1) has also been sporadically isolated from dead wild birds in Hong Kong Special Administrative Region, People’s Republic of China; however, these birds were considered dead-end hosts of viruses acquired from poultry (*3*,*4*). In April 2005, an influenza (H5N1) outbreak was detected in bar-headed geese (*Anser indicus*) at Qinghai Lake in western China (*5*). Following this outbreak, the Qinghai-like (clade 2.2) influenza virus (H5N1) lineage was detected in wild birds and domestic poultry in countries in central Asia, the Middle East, Europe, and Africa (*6*–*10*). The source of these introductions, while still debated, is likely through bird migration, although in some instances, the role of the poultry trade has not been ruled out (*6*–*12*).

The clade 2.2 influenza (H5N1) viruses continue to be detected throughout these regions; 69 human cases with 31 deaths were reported from Azerbaijan, Djibouti, Egypt, Iraq, Nigeria, Pakistan, and Turkey from January 2006 through December 2007 (*13*). Since early 2007, the Qinghai-like influenza (H5N1) lineage has continued its geographic spread and has been reported from more than 40 countries in Eurasia and Africa (*6*). The continued detection of these viruses in Africa, Europe, and the Middle East from mid-2006 onward suggests that the virus may now be endemic in these regions.

## The Study

**Figure 1:**
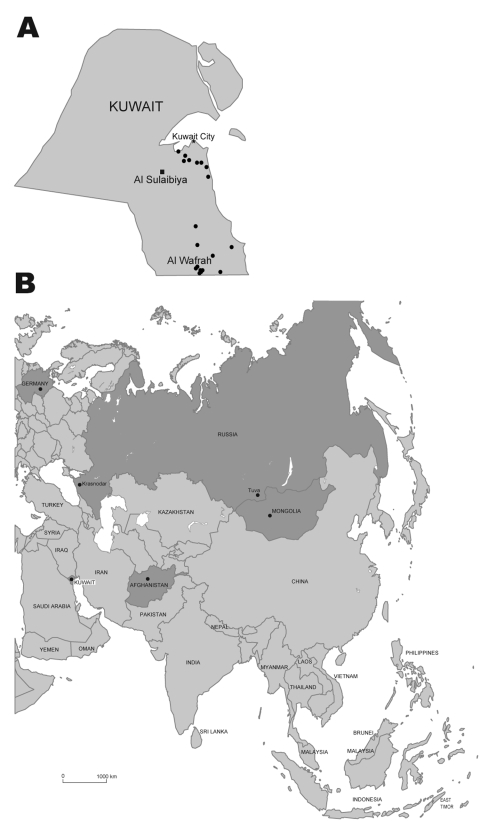
A) Kuwait, with location of subtype H5N1 virus outbreaks in 2007. Circles indicate location of farms with confirmed influenza (H5N1) infections in poultry; square indicates the Al Sulaibiya area where virus isolation was conducted. B) Eurasia, with location of subtype H5N1 isolates phylogenetically related to Kuwait isolates.

On February 13, 2007, the Public Authority for Agriculture and Fisheries of Kuwait reported the initial outbreak of influenza (H5N1) in poultry in the Al Wafrah farm area in southern Kuwait. Subsequently, 131 influenza virus (H5N1)–infected poultry were confirmed from 20 farms throughout the country (Figure 1, **panel A**). The disease resulted in high mortality rates among infected flocks, especially in the commercial broiler farms in Al-Wafrah and among poultry raised in privately owned residential homes and backyard farms. Disease control measures were implemented beginning February 18, 2007, including control of poultry movement, vaccination, disinfection of infected premises, and culling of ≈500,000 birds. The final case of subtype H5N1 was detected on April 20, 2007, and all restrictions were lifted on May 12, 2007. Kuwait was declared free of highly pathogenic avian influenza (HPAI) (H5N1) on July 21, 2007.

During these outbreaks, 20 samples were collected from small backyard farms in the Al Sulaibiya area (Figure 1, **panel A**). Among those samples, 10 throat and cloacal swabs were collected from chickens that showed signs of disease; 10 more samples were collected from internal organs (liver and spleen) of dead chickens. Seven of the 10 organ samples tested positive for subtype H5N1 by using the TaqMan Influenza A/H5 Detection Kit v1.0 on the 7500 Real-Time PCR System (Applied Biosystems, Foster City, CA, USA) according to the manufacturer’s instructions.

We sequenced the complete genome of these 7 subtype H5N1 strains isolated from poultry outbreaks in Kuwait during 2007. All sequences that were generated in this study have been deposited in GenBank (accession nos. CY029945–CY030000). To understand the developments of influenza A virus (H5N1) in Kuwait, we characterized and phylogenetically analyzed all 8 gene segments of these 7 viruses with all available influenza (H5N1) viruses previously isolated from Africa, Eurasia, Southeast Asia, and southern China, and with reference viruses belonging to each subtype H5N1 clade. Sequence assembly, editing, multiple sequence alignment, neighbor-joining, and Bayesian phylogenetic analyses were conducted as previously described (*11*).

**Figure 2:**
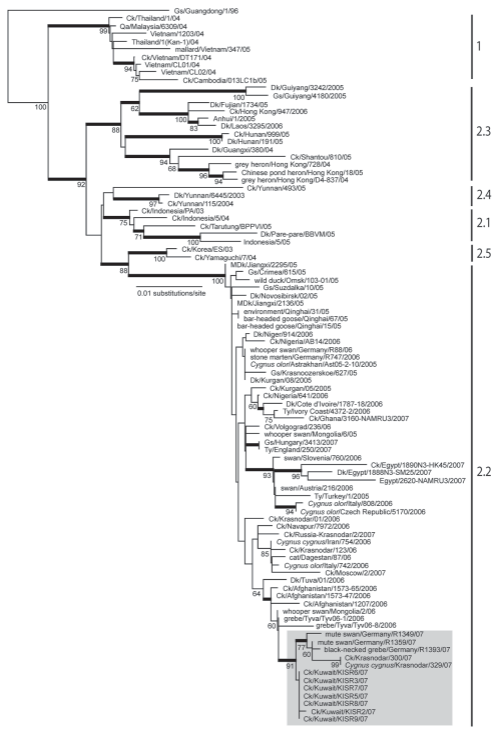
Phylogenetic relationships of the hemagglutinin (HA) gene of influenza virus (H5N1) isolates from Kuwait in 2007. Numbers at nodes indicate neighbor-joining bootstraps >60, and Bayesian posterior probabilities >95% are indicated by thickened branches. Analyses were conducted with nucleotide positions 1–963 of the HA gene. The HA tree was rooted to Gs/Guangdong/1/1996. Labels to the right of the tree refer to World Health Organization (H5N1) clade designations (*14*). Ck, chicken; Dk, duck; Gs, goose, MDk, migratory duck; Qa, quail; Ty, turkey.

Phylogenetic analysis of the hemagglutinin (HA) genes showed that all 7 subtype H5N1 isolates were derived from the Goose/Guangdong-like lineage and clustered together with other Qinghai-like (clade 2.2) viruses (Figure 2). The Kuwait isolates were most closely related to viruses from Germany and Krasnodar, in southwest Russia, which were also isolated in 2007 (Figure 1, **panel B**). Those viruses were mostly isolated from wild bird species (swan and grebe), although a single isolate was from chicken in Krasnodar. This group of viruses was in turn related to 2006 isolates from diverse geographic areas such as Afghanistan, Mongolia, and Siberian Russia (Figure 1, **panel B**). Phylogenetic analyses of the neuraminidase gene and all internal gene segments (data not shown) show that all of the viruses belong to subtype H5N1, genotype Z, and maintain phylogenetic relationships similar to the HA tree.

The HA protein of all 7 isolates maintained the motif of multiple basic amino acids (QGERRRKKR/G) at the HA-connecting peptide, a feature that is characteristic of HPAI virus. The receptor-binding pocket of HA1 retains Gln 222 and Gly 224 (H5 numbering) that preferentially binds avian-like α2,3-NeuAcGal linkages. However, a single Glu212Lys substitution occurred in the HA receptor binding site in all 7 Kuwait isolates, which has also been observed in all clade 2.2 influenza (H5N1) viruses characterized to date. The biological implications of this mutation remain to be investigated. None of the isolates had mutations in the M2 ion channel or the neuraminidase, conferring resistance to amantadine and oseltamivir, respectively. All isolates possessed Lys at position 627 of the PB2 gene, which is associated with increased virulence in mammals and is present in all known clade 2.2 viruses. Other virulence mutations were not recognized in any of the viruses characterized in this study.

Antigenic characterization of a representative virus from Kuwait (Ck/Kuwait/KISR2/2007) was conducted as previously described (*11*). These results demonstrate close antigenic relationship of Ck/Kuwait/KISR2/2007 to BHG/Qinghai/1A/2005 (Table), the prototype clade 2.2 virus, and a vaccine candidate virus that was isolated during the HPAI (H5N1) outbreak in wild birds in Qinghai Lake, China, in 2005 (*5*).

**Table:** Antigenic analysis of influenza viruses (H5N1) by hemagglutinin inhibition test, 2007*

Virus	Clade†	Anti-VNM/1203‡	Anti-IDN/5	Anti-CDC357	Anti-QH/1A	Anti-Anhui/1
Vietnam/1203/2004	1	**160**	160	40	40	40
Indonesia/5/2005	2.1	<40	**640**	640	40	80
Indonesia/CDC357/2006	2.1	40	1,280	**1,280**	80	160
BHG/Qinghai/1A/2005	2.2	80	640	320	**160**	640
Dk/Laos/3295/2006	2.3.4	40	320	80	40	320
Ck/Kuwait/KISR2/2007	2.2	<40	640	160	320	<40

*BHG, bar-headed goose; Dk, duck; Ck, chicken. **Boldface** numbers indicate titers to prototype viruses. †Based on the World Health Organization (H5N1) nomenclature system (*14*). ‡Ferret antisera dilution started at 1:40.

## Conclusions

The results of this study confirm that clade 2.2 HPAI (H5N1) viruses were responsible for the poultry outbreaks recorded in Kuwait in early 2007. Notably, the viruses from Kuwait are most closely related to other 2007 subtype H5N1 isolates from Germany and Russia, but not to other 2007 isolates from Egypt, England, Ghana, and Hungary for which data are available (Figure 2). Furthermore, none of the current isolates from Europe or the Middle East has a close phylogenetic relationship with clade 2.2 isolates from China in 2005, although data on recent subtype H5N1 isolates from northern China are lacking (*5*,*12*). These relationships, along with reemergence of genetically similar viruses in widely distant geographic locations such as Germany, Krasnodar, and Kuwait (Figure 1, **panel B**), indicate that clade 2.2 influenza (H5N1) viruses may have become endemic in wild birds in central or eastern Asia (including Siberian Russia), from where they have been repeatedly introduced to Europe and the Middle East. Although it remains unclear in which hosts these viruses are maintained, the geographic distribution of closely related viruses suggests that migratory bird species are likely acting as vectors. Also, continued endemicity of clade 2.2 viruses in parts of Eurasia may result in the diversification of the virus in different geographic areas, as has been seen for subtype H5N1 lineages in eastern and Southeast Asia (*11*). Therefore, systematic surveillance in poultry and wild bird populations will be an important tool for tracking the evolution of clade 2.2 influenza (H5N1) viruses in this region.
